# Carbohydrate Metabolism Differentiates *Pectinatus* and *Megasphaera* Species Growing in Beer

**DOI:** 10.3390/microorganisms12102045

**Published:** 2024-10-10

**Authors:** Manuel J. Arnold, Stefan W. Ritter, Matthias A. Ehrmann, Yohanes N. Kurniawan, Koji Suzuki, Thomas M. Becker, Wolfgang Liebl

**Affiliations:** 1 Chair of Microbiology, Technical University of Munich, 85354 Freising, Germany; 2 Chair of Brewing and Beverage Technology, Technical University of Munich, 85354 Freising, Germany; 3 Asahi Quality and Innovations, Ltd., 1-1-21 Midori, Moriya 302-0106, Ibaraki, Japan

**Keywords:** beer spoilage, *Pectinatus* spp., *Megasphaera* spp., carbohydrate metabolism, volatile fatty acids

## Abstract

Obligate anaerobic beer spoilage bacteria have been a menace to the brewing industry for several decades. Technological advances in the brewing process aimed at suppressing aerobic spoilers gave rise to problems with obligate anaerobes. In previous studies, the metabolic spectrum of *Pectinatus* and *Megasphaera* species has been described, but their metabolism in the beer environment remains largely unknown. We used high-performance anion exchange chromatography with pulsed amperometric detection (HPAEC-PAD) and headspace solid-phase microextraction–gas chromatography–mass spectrometry (HS-SPME-GCMS) to further characterize beer spoiled by 30 different strains from six beer-spoiling species of *Pectinatus* and *Megasphaera* (*P. cerevisiiphilus*, *P. frisingensis*, *P. haikarae*, *M. cerevisiae*, *M. paucivorans*, and *M. sueciensis*). We detected differences in carbohydrate utilization and the volatile organic compounds (volatilome) produced during beer spoilage by all six species. We were able to show that glycerol, one of the basic components of beer, is the common carbon source used by all strains. It appears that this carbon source allows for anaerobic beer spoilage by *Pectinatus* and *Megasphaera* despite the spoilage-preventing intrinsic barriers of beer (iso-α-acids, ethanol, low pH, scarce nutrients); thus, extrinsic countermeasures are key for prevention.

## 1. Introduction

Beer spoilage through bacteria has been an issue for many years [1,2,3,4]. This not only poses an economic threat to companies but also causes a possible negative perception of a brand due to the obnoxious flavors caused by them [5,6,7]. The composition of beer represents a harsh challenge to most microorganisms because of several intrinsic barriers [8]. The isomerized α-acids derived from hops as well as the ethanol content and the low pH confer antimicrobial properties to the final product. Beer spoilage bacteria are known to have a series of properties to circumvent these obstacles and grow nonetheless [9,10,11,12,13,14,15].

Over the past decades, consistent improvement in the brewing process has increased the importance of anaerobic beer spoilers. By decreasing the dissolved oxygen in all stages of the process, the wort and final beer have become less susceptible to aerobic spoilers while giving anaerobic spoilage bacteria an edge over them [16]. Anaerobic spoilage bacteria tend to cause beer spoilage at the fermentation or maturation stage. At this point of the process, raw materials as well as time and effort have already been applied to the production. This causes the spoilage by obligate anaerobes to have a higher negative financial impact than that by aerobic spoilers [17,18].

The most dreaded obligate anaerobic spoilers are representatives of the Gram-negative-staining genera *Megasphaera* and *Pectinatus.* The genus *Megasphaera* comprises several species of strictly anaerobic, non-motile cocci, which have been isolated from the rumen of livestock, human feces and intestines, and spoiled beer [19,20,21,22]. Brewery-associated *Megasphaera* spp. are *M. cerevisiae*, *M. paucivorans*, and *M. sueciensis*, which are 0.4–2 µm in size and are not spore-forming. Their original habitat remains unknown as of yet [19,21]. *Pectinatus*, a genus first described over forty years ago by Lee et al. [23] and emended by Schleifer et al. [5], consists of motile, non-spore-forming rods initially isolated from spoiled beer and salty wastewater [5,23,24,25]. Their size is around 0.4–0.9 × 2.0–32 µm, and the species *P. cerevisiiphilus*, *P. frisingensis*, and *P. haikarae* are the only representatives isolated from beer or the brewery environment [5,21,23,26].

These spoilage bacteria must be able to cope with nutrient scarcity, alongside their toolset of defense mechanisms against stressors. While their ability to withstand beer-associated stress has been the topic of research for years, the current study is the first to address their specific carbohydrate metabolism in beer. In this study, we focused on obligate anaerobic spoilers from six different species of the genera *Pectinatus* and *Megasphaera*. The aim was to gain deeper insight into the carbohydrate metabolism of 30 different strains during growth on lager beer by measuring the residual sugars and the volatiles produced after spoilage using a high-performance anion exchange chromatography system equipped with a pulsed amperometric detector (HPAEC-PAD) and headspace-solid phase microextraction–gas chromatography–mass spectrometry (HS-SPME-GCMS). We intended to bridge the gap in the descriptions of obligate anaerobic beer spoilers from the genera *Pectinatus* and *Megasphaera* by further examining their metabolism in the beer environment as this has been neglected by research so far.

## 2. Materials and Methods

### 2.1. Bacterial Strains and Cultivation

Bacterial strains were taken from our in-house TMW (Technische Mikrobiologie Weihenstephan) strain collection and are listed in Table 1. Cultivation was carried out at 30 °C under anaerobic conditions using modified PYG Medium (5 g/L trypticase peptone, 5 g/L peptone, 10 g/L yeast extract, 5 g/L beef extract, 5 g/L glucose, 2 g/L K_2_HPO_4_, 20 mg/L MgSO_4_ × 7 H_2_O, 40 mg/L KH_2_PO_4_, 1 mL/L Tween 80, 80 mg/L NaCl, 10 mg/L CaCl_2_ × 2 H_2_O, 0.40 g/L NaHCO_3_, 250 mg/L Resazurin, 1 mL/L NBB-C, 1 µL/L Vitamin K_1_, 5 mg/L Haemin solution, 0.5 g/L Cysteine-HCl × H_2_O). For this procedure, Hungate tubes were filled with forming gas (5% H_2_, 95% N_2_, Westfalen AG, Münster, Germany) prior to autoclaving and filling via a syringe through the septum. The inoculation was also executed via a syringe.

### 2.2. Beer Spoilage Assay

For the beer spoilage assay, pale lager beer (5.1% ABV, 21 IBU, pH 4.3) was roughly degassed by inversion in bottles with twice the volume of the liquid and subsequently degassed in an ultrasonic water bath for 30 min. After an additional 30 min in a 60 °C water bath, the beer was sterile-filtered (500 mL Rapid-Flow Bottle Top Filter, 0.2 µm aPES membrane, Thermo Fisher Scientific Inc., Waltham, MA, USA), modified, and distributed among sterile Hungate tubes under a clean bench. All strains were inoculated in biological triplicates to a starting concentration of 1 × 10^5^ CFU/mL of cells from an overnight culture and anaerobically incubated at 30 °C whilst turbidity was monitored using a densitometer (Grant-Bio DEN-1B, Grant Instruments Ltd., Royston, UK). After a period of 23 days, the final pH of all the samples was measured, and then, the samples were transferred into reaction tubes, centrifuged, and the supernatant taken for analysis. All strains showed growth in the lager beer used (Figure S1).

### 2.3. Ion Chromatography

#### 2.3.1. Chemicals

The analytical standards, including glucose (CAS 50-99-7), fructose (CAS 57-48-7), sucrose (CAS 57-50-1), maltose (CAS 6363-53-7), maltotriose (CAS 1109-28-0), xylose (CAS 58-86-6), ribose (CAS 50-69-1), arabinose (CAS 10323-20-3), and glycerol (CAS 56-81-5), and the internal standard for the carbohydrate analysis, 2-deoxy-D-glucose (CAS 154-17-6), were purchased from Sigma-Aldrich (Steinheim, Germany). Maltulose (CAS 207511-09-9) was purchased from Genaxxon bioscience (Ulm, Germany). The 50% sodium hydroxide solution (CAS 1310-73-2) for the eluent and methanol (CAS 67-56-1) used for dilution in the carbohydrate analysis were obtained from VWR International (Darmstadt, Germany). The water used for dilution and buffers was membrane-filtrated with a micropore water purification system (Thermo Fisher Scientific Inc., Waltham, MA, USA).

#### 2.3.2. Carbohydrate Analysis

Carbohydrate analysis was performed using a high-performance anion exchange chromatography system equipped with a pulsed amperometric detector (HPAEC-PAD). This system was composed of an ICS AS/AP autosampler, an ICS 5000 DC column compartment, and an ICS 5000 DP pump module (all from Thermo Fisher Scientific Inc., Waltham, MA, USA). As the stationary phase, a Dionex CarboPac PA10 column (2 mm × 250 mm) and a Dionex CarboPac PA10 guard column (2 mm × 50 mm) were utilized for all measurements (both Thermo Fisher Scientific Inc., Waltham, MA, USA). The PAD cell consisted of a titanium cell body, a disposable gold working electrode, and a silver/silver chloride pH reference electrode. The detector settings were as follows: 0.1 V at 0.00 s; 0.1 V at 0.40 s; −2.0 V at 0.41 s; −2.0 V at 0.42 s; 0.6 V at 0.43 s; −0.1 V at 0.44 s; and −0.1 V at 0.50 s. Data acquisition was performed with 5 data points per second, and data processing was performed with Chromeleon 7.2 software from Thermo Fisher Scientific Inc. (Waltham, MA, USA).

For the mobile phase, two eluents, 250 mM sodium hydroxide (A) and HPLC-grade water (B), were utilized. Both eluents were degassed by ultrasonic treatment for at least 5 min. Once attached to the HPAEC device, the eluents were kept under a pressurized inert helium atmosphere. The flow rate was set to 0.25 mL/min, and the gradient setting was 20% A at 0 min, 20% A at 13 min, 97% A at 14 min, 97% A at 27 min, 99% A at 28 min, 99% A at 39 min, and 20% A at 41 min, followed by 4 min of equilibration at 20% A. For changes in the eluent ratio, a linear curve (curve 5 in Chromeleon 7.2) was applied.

The method was calibrated by injecting seven different concentrations of the analytical standards, including glucose, fructose, saccharose, maltose, maltotriose, xylose, ribose, arabinose, glycerol, and maltulose, in two replicates. The coefficients of correlation (R^2^) were >0.99 for all compounds, and the linear ranges are listed in Table S2.

The method validation included a recovery analysis, the determination of the limit of detection (LOD) and limit of quantification (LOQ), and a repeatability test. For the recovery analysis, a standard mixture containing standards for all analytes was mixed with the beer (a commercial product that was used in this study for the fermentation experiments) and fermented beer (pooled samples fermented with all individual strains used in this study) matrices. This was performed in sextuplicate for each matrix. The recovery rates were 83–109% for beer and 86–110% for fermented beer (see Table S2). The LOD and LOQ values were determined from the fluctuations in the baseline and the slope of the calibration curve, as described by Ritter et al. [27]. The LOD values were between 0.11 and 1.57 mg/L, and the LOQ values were between 0.33 and 4.76 mg/L (see Table S2). For the repeatability test, a standard mixture was injected 10 times over a period of approx. 24 h. All the measured concentrations fluctuated within 5% of the average value of the respective analyte, and no trends were observable. The shifts in the retention times were all less than 0.3%.

The sample preparation included dilution in 50% (*v*/*v*) methanol and filtration through dead-end syringe filters (0.45 µm pore size, obtained from Macherey-Nagel, Düren, Germany) prior to injection.

### 2.4. Headspace SPME-GCMS Measurements

Headspace solid-phase microextraction–gas chromatography–mass spectrometry (HS-SPME-GCMS) was carried out on a gas chromatograph (GC) Trace 1310 (Thermo Scientific Inc., Waltham, MA, USA) directly coupled to an ISQ-7000 quadrupole-mass spectrometer (Thermo Scientific Inc., Waltham, MA, USA). The GC device was equipped with a TG-5MS column (60 m × 0.25 mm; film thickness of 0.25 µm; Thermo Scientific Inc., MA, USA), and the carrier gas was helium with a constant flow rate of 1.25 mL/min. The injector was heated to 250 °C, the transfer line to 250 °C, and the ion source to 200 °C. The starting temperature of 60 °C was maintained for 4 min, followed by heating by 5 °C/min to 200 °C and subsequently 10 °C/min to 250 °C, where it was held constant for 3 min. The mass spectrometer was operated in EI mode with 70 eV, and the detection range was 3–350 Th. The samples were analyzed in duplicates by using 5 mL of sample in a 20 mL headspace vials and capped with a bimetal lid (8 mm hole, PTFE septum, Wagner & Munz, München, Germany). SPME extraction was performed using an SPME-ARROW Fiber Tool (PAL) and a Triplus RSH autosampler (Thermo Scientific Inc., Waltham, MA, USA). The applied fiber was a stable flex ARR11-DVB/CWR/PDMS (1.1 mm; phase of 20 mm; phase diameter of 120 µm; Supelco, Bellafonte, PA, USA), which was heated to 270 °C for 30 min before initial use and before and after every analysis for 1 min at 250 °C while being purged with helium. The samples were extracted by introducing the SPME fiber to the sample’s headspace under permanent stirring (40 °C; 100 rpm) for 30 min. Desorption took place by inserting the fiber for 1 min into the injector at 250 °C. Peak detection was performed using Xcalibur 4.1 software (Thermo Scientific Inc., Waltham, MA, USA) and the NIST 11 spectral library. For qualitative analysis, we used a method by Schnaitter et al. [28] with the modification that the obtained peak areas were analyzed by applying ANOVA and a Dunnett comparison to reveal significant rises in signal magnitude compared with the control (*p* ≤ 0.05).

### 2.5. pH Measurements

After ending the incubation of the replicates in Hungate tubes, aliquots were taken by syringe and transferred into a microwell plate. The pH was measured within the wells of the microplate using a pH meter (SevenGo, Mettler Toledo AG, Zurich, Switzerland).

### 2.6. Statistics and Figures

ANOVA calculation and subsequent Dunnett comparison were carried out in R (v4.3.3; R Core Team 2023) using the packages DescTools (v0.99.50) [29] and report (v0.5.7.12) [30]. Figures were created using R in combination with the packages ggplot2 (v3.4.4) [31], heatmaply (v1.5.0) [32], and factoextra (v1.0.7) [33] as well as OriginPro 2021 (v9.8.0.200; OriginLab Corporation 1991–2020).

### 2.7. Availability of Data and Materials

The genomes of type strains used to search genes are available on the NCBI website under their respective accession numbers as follows: *Megasphaera cerevisiae* DSM 20462 (NZ_FUXD01000109.1) and *Megasphaera paucivorans* DSM 16981 (NZ_FNHQ01000075.1).

## 3. Results

### 3.1. Residual Carbohydrates in Beer

To determine the initial nutrients available for all spoilers, we determined several compounds in the commercial lager beer used in this study via ion chromatography. Several sugars and sugar alcohols were assessed to depict which were present in beer and which were eventually utilized by the anaerobic spoilage bacteria tested (Table S1).

**Figure 1:**
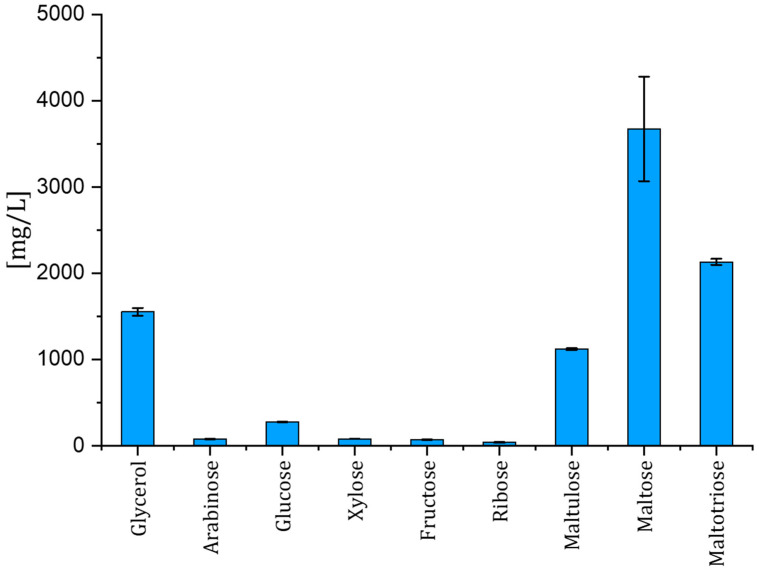
Composition of sugars in the utilized commercial lager beer from triplicates. Error bars indicate the standard deviation for each compound.

This resulted in focusing on the following carbon sources: glycerol, arabinose, xylose, fructose, ribose, maltulose, maltose, and maltotriose, of which glycerol, maltulose, maltose, and maltotriose proved most abundant. Their concentrations as well as the standard deviation in the lager beer were determined and portrayed in Figure 1. Maltose showed a relatively high standard deviation in comparison with the other substrates; therefore, we measured 13 commercially available lager beers from large breweries to assess the deviation in all substrates at a wider scope.

The sugars at low abundance in lager beers showed the following deviations among the tested beers: arabinose (33.8 ± 8.2 mg/L), glucose (93.7 ± 35.5 mg/L), xylose (53.6 ± 22.5 mg/L), fructose (37.5 ± 24.0 mg/L), and ribose (50.7 ± 7.8 mg/L). Compounds at high concentrations such as glycerol (1.41 ± 0.20 g/L), maltulose (1.16 ± 0.22 g/L), maltose (0.60 ± 0.93 g/L), and maltotriose (1.23 ± 0.94 g/L) showed a comparatively higher deviation. Maltose and maltotriose showed a high divergence among all the beers tested. It has to be mentioned that the mean total sugar concentrations of all thirteen beer samples showed a deviation of 4.67 ± 1.96 g/L, while the beer used in our study showed a slightly higher concentration of 6.16 ± 1.48 g/L among different batches. The lager beer used in our work showed a high deviation in the concentrations of maltose (1.27 ± 1.24 g/L) and maltotriose (1.81 ± 0.46 g/L) between batches as well. Measurements of the sugars and glycerol in the control replicates of the lager beer used in the spoilage assay showed a consistent profile of these compounds in the samples (Figure 1). The determination of glucose was not possible because of the possible carryover of glucose from the medium of the preculture used for inoculation. Because of its low abundance, glucose was not considered a major contributor.

### 3.2. Carbohydrate and Glycerol Utilization and Odor Compound Production by Megasphaera *spp.* Strains during Lager Beer Spoilage

Lager beer spoilage assays were carried out in triplicate with the *Megasphaera* spp. and *Pectinatus* spp. strains as described in the Section 2. The degradation of sugars and glycerol varied significantly between the different species from the different genera.

Further examining the strains of each genus revealed a diverse behavior in the usage of the substrates present in beer by strains of the genus *Megasphaera* (Table 2). All *Megasphaera* strains showed a slight but significant degradation of glycerol by an average of 19%. Arabinose, on the other hand, was not a carbon source generally used in this genus, with only three strains significantly reducing its amount in the medium. *M. cerevisiae* strains TMW 2.2484 and TMW 2.2485 were able to degrade most of the arabinose (77% and 79%, respectively), while the type strain DSM 20462^T^ removed it from the medium completely. None of the strains utilized xylose, but all of them degraded fructose completely with the exception of *M. cerevisiae* TMW 2.2482 and *M. sueciensis* DSM 17042^T^; while the first proved incapable of metabolizing fructose, the latter decreased it significantly but not entirely. Ribose was utilized by only three strains (DSM 20462^T^, TMW 2.2482, and DSM 17042^T^) and merely to a very low extent. None of the strains were able to degrade maltulose or maltose, and maltotriose was consumed only by a small amount by strains TMW 2.2480, TMW 2.2484, and TMW 2.2485.

All representative strains from the genus *Megasphaera* showed a distinct output of odors (Table 3). Among the compounds exclusively found after the growth of *Megasphaera* spp. in lager beer were n-butanol, butyric acid, and caproic acid as well as ethyl nicotinoate. A compound solely produced by *M. cerevisiae* strains was 2-methylbutanoic acid, whereas 3-methylbutanoic acid was produced by all *Megasphaera* spp. strains. Strains of the species *M. paucivorans* showed the formation of 4-vinylphenol and citronellol as an exclusive trait, while α-terpineol was only produced by *M. sueciensis.*

### 3.3. Carbohydrate and Glycerol Utilization and Odor Compound Production by Pectinatus *spp*. Strains during Lager Beer Spoilage

The twenty strains from the genus *Pectinatus* showed a much more diverse metabolic behavior (Table 4) than the *Megasphaera* strains.

All investigated strains from the genus *Pectinatus* could utilize the glycerol present in lager beer as a carbon source, with the majority depleting it entirely. The highest residue in glycerol was measured in beer spoiled by TMW 2.2490, which left 12% of the substrate behind. Arabinose utilization showed a more diverging pattern. While all *P. frisingensis* strains proved a high capability to metabolize it, the strains belonging to other *Pectinatus* species differed vastly in their ability to use it. Two of four *P. cerevisiiphilus* strains degraded arabinose entirely, whereas the other two only slightly degraded arabinose. Within the species *P. haikarae*, the type strain utilized arabinose completely, while the other two *P. haikarae* strains were the only strains of the genus that did not significantly degrade this pentose. Only six strains were able to metabolize xylose by a significant amount, of which four belonged to the species *P. cerevisiiphilus* (TMW 2.1494, DSM 20467^T^, TMW 2.2465, TMW 2.2492) and one to *P. haikarae* (DSM 16980^T^). *P. frisingensis* TMW 2.2474 decreased the xylose content of the medium completely but was the only 1 of 13 *P. frisingensis* strains to degrade xylose significantly. All strains of all three *Pectinatus* species showed high capacity for fructose utilization. While all *P. cerevisiiphilus* and *P. frisingensis* strains depleted the available fructose completely, the *P. haikarae* strains just partially utilized between 40% and 80% of this monosaccharide. Ribose degradation was quite variable among the *Pectinatus* strains. Of the type strains of *P. cerevisiiphilus* and *P. frisingensis*, the former reduced the ribose by about one-third, while the latter degraded this pentose completely. On the other hand, several strains of these two species neglected ribose, as well as all of the *P. haikarae* strains. Some *P. frisingensis* strains even showed an increase in ribose (TMW 2.1490, TMW 2.1493, TMW 2.2467, and TMW 2.2469). The utilization of the disaccharide maltulose was also variable among the strains investigated.

The *P. frisingensis* strains utilized between 6% and 68% of the maltulose present in the medium, whereas strains of the other two species, i.e., *P. cerevisiiphilus* and *P. haikarae*, only consumed between 9 and 17% and 11 and 18%, respectively, of this disaccharide within their species. All three species yield strains that did not significantly utilize maltulose (TMW 2.1490, DSM 20467^T^, DSM 16980^T^, and TMW 2.2469). The most abundant sugar in beer, i.e., maltose, was significantly utilized by all *Pectinatus* strains. The strains of the species *P. cerevisiiphilus* consumed between 32% and 42%, the representatives of the species *P. haikarae* consumed between 41% and 43%, and the strains of *P. frisingensis* degraded between 38% and 94% of the maltose present. Maltotriose served as another reliable carbon source, being significantly decreased by most strains by approximately 15% and by *P. haikarae* strains by 10%, with the exception of only a few strains from the species *P. frisingensis* (TMW 2.1503, DSM 6306^T^, TMW 2.2471, TMW 2.2479) and *P. cerevisiiphilus* (DSM 20467^T^).

All *Pectinatus* spp. strains produced a flavor profile distinct from that of their *Megasphaera* counterparts (Table 5). They all produced propanoic acid and ethyl propanoate. Also, all *Pectinatus* strains except one *P. haikarae* strain (TMW 2.1496) produced isobutyric acid and geraniol, and most strains produced acetic acid with the exception of a few *P. cerevisiiphilus* (DSM 20467^T^, TMW 2.2465) and *P. haikarae* (TMW 2.1496) strains that did not produce acetic acid.

A compound that was characteristic of *P. frisingensis* was α-terpineol. It was only found in beer spoiled by strains of this species with the exception of the beer spoiled by *M. sueciensis*. Another monoterpenoid that was only produced by *P. frisingensis* was nerol, even though not all strains produced it. The *P. haikarae* strain TMW 2.2490 uniquely produced 2-methylbutyl octanoate, 3-methylbutyl octanoate, 4-vinyl guajacol, methyl-E-geranate, and ethyl-4-methyl pentanoate. Furthermore, this strain together with another *P. haikarae* strain, DSM 16980^T^, were the only ones to produce methional.

### 3.4. Change in pH after Spoilage

**Figure 2:**
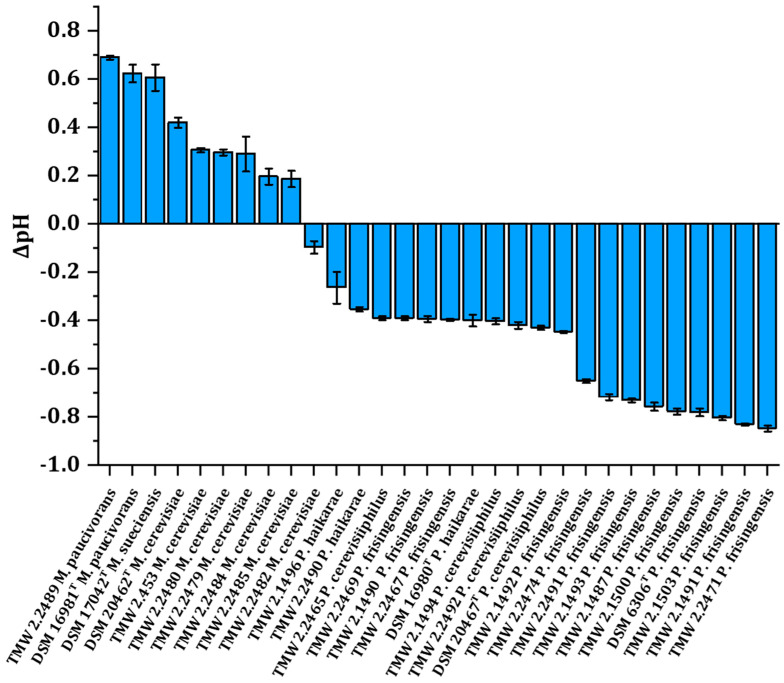
Change in pH in triplicates of lager beer spoiled by the respective strains’ initial lager beer pH of 4.3. Strains are sorted by the difference in pH compared with the control beer. A superscript T indicates the respective type strain, and error bars represent the standard deviation.

To help further contextualize the results obtained, in particular concerning the utilization of residual carbohydrates and glycerol in lager beer by strains of the genera *Megasphaera* and *Pectinatus*, we conducted pH measurements in all spoiled beers to assess the final pH after spoilage. The change in pH seemed to be related to the genus, as *Megasphaera* spp. strains generally increased the pH, whereas members of the *Pectinatus* genus decreased the pH (Figure 2). The *M. paucivorans* and *M. sueciensis* strains had a higher capacity to elevate the pH in comparison with their *M. cerevisiae* counterparts. The strains that decreased the pH the furthest all belong to the species *P. frisingensis*.

## 4. Discussion

### 4.1. Residual Carbohydrates in Beer

The measured constitution of the lager beer used for the beer spoilage assay was similar to the previously described contents of beer [34,35]. While the amounts of maltose are in a reasonable range, the observed standard deviation in the measurements was rather high [34,35,36]. This might be process-related and could be coupled to yeast performance since yeasts are harvested and used several times depending on the yeast handling of the brewery. While the attenuation of yeast during fermentation has been the topic of research in the past years, the view of its dependency on repitching is controversial [37,38,39,40].

The intrinsic barriers of beer (ethanol, low pH, and iso-α-acids) are key for the prevention of microbial growth and therefore spoilage. Beer spoilage bacteria possess mechanisms to tolerate those stressors, but in order to grow, they need suitable carbon sources. By measuring the residual carbohydrates and glycerol in spoiled beer, we wanted to reveal the carbon sources targeted by beer-spoiling *Pectinatus* spp. and *Megasphaera* spp. strains. Glycerol is among the three main components introduced by yeast fermentation next to ethanol and CO_2_. Its content measured in the present work is in line with previous findings [41,42]. The “smoothness” it imparts on the taste of the final beverage is widely acknowledged [43,44]. While glycerol helps the yeast retain cellular function under osmotic stress [45,46], it is also a consequence of sustaining the redox potential by oxidizing excess NADH via glycerol synthesis [47]. Since glycerol is generally present in beer, it may serve as an important substrate for the growth of all the spoilers examined in this study.

Another sugar that proved to be important for the discrimination of obligate anaerobic beer spoilers was maltulose. The occurrence of maltulose has been known for about seventy years now [48,49]. It is formed as a by-product during starch saccharification in the commercial production of glucose [48,49,50,51] or directly from maltose in a pressurized buffer system [51]. In food quality control, maltulose serves as a quality criterion [52,53,54]. Aside from its selective production it most likely emerges during mashing and boiling from the thermal load in the brewing process, eventually residing in the cast wort. Maltulose has been shown to be metabolized by a wide variety of yeast strains but not necessarily by all strains [55]. Therefore, residual maltulose is a common constituent in finished beer and an important carbohydrate for some of the obligate anaerobic spoilers investigated in this study. Strikingly, the role of maltulose as a carbon source for beer spoilage bacteria has been neglected so far. Since there are no maltulose-specific enzymes described in the literature, its degradation might be linked to maltose degradation and a cross-reactivity of the respective catabolic enzymes towards maltulose [56,57].

The utilization of maltotriose by yeasts is determined by its uptake, which is regulated by the transporters responsible for the intracellular uptake of maltose [58,59,60]. This varies among yeast strains because of the different activity of transporters and therefore creates alternating concentrations of the trisaccharide, representing another useful carbohydrate source if degradable by spoilage strains [59].

### 4.2. Carbohydrate and Glycerol Metabolism by Megasphaera *spp*.

The sugars and sugar alcohols metabolized by *Megasphaera* spp. only partially coincide with what is described in the literature [19,21,22]. A reason for this could be the use of different strains as the strains described in previous reports are mostly type strains, which were also applied in this study in addition to strains not previously described. Furthermore, this study differs from previous publications by application of an HPAEC-PAD to quantify the residual sugars whereas previous studies applied growth experiments with the respective carbohydrates as a sole source [19,21,22]. Referring to the overview of carbon sources utilized by *Megasphaera* spp. (Table 2), it becomes apparent that the strains mainly metabolize glycerol, while the decrease in maltose was not significant. Previous investigations that studied the ability to use maltose [19,21] did not include such a large variety of strains as that used by us and applied a qualitative approach that did not allow for observing minor differences in maltose during spoilage.

Despite only employing glycerol, the strains of this genus all grew, suggesting additional carbohydrate-independent metabolic routes, which is also suggested by the increase in pH of the spoiled beer (Figure 2). Several types of decarboxylases are known to be involved in the acid stress response of microorganisms [61,62,63] but have so far not been described in beer-spoilage strains of the genus *Megasphaera.* We hypothesize that beer-spoiling members of this genus could harbor decarboxylases metabolizing free amino acids, e.g., arginine, glutamate, lysine, or glutamine, that are known to be present in wort and beer [42]. A review of the available genomes of the type strains at the National Center for Biotechnology Information (NCBI) revealed arginine decarboxylases via annotation or BLAST search using a known amino acid sequence, supporting our assumptions (NZ_FUXD01000109, LOCUS_10910; NZ_FNHQ01000075, LOCUS_02800). In addition, an ornithine decarboxylase was found in both type strain genomes (NZ_FUXD01000109, LOCUS_03460; NZ_FNHQ01000075, LOCUS_07150, LOCUS_03910). Arginine as well as ornithine are common ingredients in beer [64,65], and it can be hypothesized that additional ornithine could result from arginine decarboxylation and therefore further fuel the observed alkalization. Perhaps the higher extent to which *M. paucivorans* alkalized the beer during incubation correlates with a higher abundance of decarboxylases in order to combat the acid stress posed by beer, but this remains to be studied in more detail.

The odors solely produced by *Megasphaera* spp. are in line with what has been reported in the literature [19,20,21,22]. While there is no report about the production of n-butanol so far, butyric acid is known to be the major fermentation product of the type species of the genus *Megasphaera*, i.e., *M. elsdenii* [22]. While the metabolism of *M. elsdenii* is known to produce mainly butyric acid from glucose, we were able to verify the literature by showing that the beer-spoiling species of *Megasphaera* all are incapable of using glucose while still producing butyric acid [19,20,21,22]. We were also able to validate caproic acid as a common volatile fatty acid among the beer-spoiling representatives of the genus *Megasphaera* [19,21]. In addition, we were able to detect compounds previously not reported to be produced by strains of the genus, i.e., ethyl nicotinoate and 3-methylbutanoic acid, which were found with the strains of all species, i.e., 2-Methylbutanoic acid only for *M. cerevisiae* strains, 4-vinylphenol and citronellol solely for *M. paucivorans*, and α-terpineol as an exclusive trait of *M. sueciensis*. The production of H_2_S reported in the literature [21] could not be measured because of the detection range, which served the purpose of excluding noise signals from air but, in this case, also excluded H_2_S.

### 4.3. Carbohydrate and Glycerol Metabolism by Pectinatus *spp*.

The metabolic spectrum of *Pectinatus* spp. has been described to some degree, but it lacks the beer and wort-specific perspective. There is a clear superiority of *P. frisingensis* over other *Pectinatus* sp. in terms of the metabolic versatility in (partially) utilizing glycerol and carbohydrates present in beer (Table 4). While the ability of *P. frisingensis* and *P. cerevisiiphilus* to metabolize glycerol, arabinose, xylose, fructose, and ribose has been mentioned [5], substrate utilization remains unclear for *P. haikarae* for all tested sugars except xylose [21]. The good growth on PYF reported by Juvonen and Suihko (2006) [21] suggests that the species can utilize fructose, which coincides with our results. Maltose was described as a carbon source for several strains of *P. frisingensis*; however, only the type strain and one other strain (DSM 6306^T^ and TMW 2.2469) were part of our study, and their results are in agreement with previously reported results [5]. Furthermore, our results are in line with the results of a publication that used genomic prediction, which stated that *Pectinatus* strains are able to use fructose, glycerol, and ribose [66] even though not all did. Another finding from this paper was the presence of xylose transporters in the genomes despite the prediction that beer-spoiling *Pectinatus* spp. strains were not able to metabolize xylose. Our results are in contrast to the reported prediction by Kramer et al. yet in line with the literature as they showed a significant decrease in xylose in all *P. cerevisiiphilus* strains, in two *P. haikarae* strains, and in one *P. frisingensis* strain [5,21,66]. All other strains of *P. frisingensis* did not metabolize xylose. We observed a significant increase in xylose among this species ranging from 7 to 30%. A possible explanation for this result could be the degradation of arabinoxylan-releasing xylose units while utilizing the arabinose units. The abundance of arabinoxylan in several beer styles has been shown [67,68], but degradation has not been described so far, and this remains to be further investigated. The increase in D-ribose in some *P. frisingensis* strains might be related to the production of pentose by the strains, as reported for other microorganisms [69,70,71]; however, further experiments are needed to study this observation in more detail. In addition to this, we showed that all species of the genus *Pectinatus* were able to use maltose significantly, which contradicts previously reported cases [21,66] but aligns with others [23]. Maltulose and maltotriose, on the other hand, have not been mentioned in the literature so far but proved to be a useful carbon source for several members of the genus even though not all strains showed a significant decrease. The superior growth in beer of *Pectinatus* spp. over *Megasphaera* spp. seen in the experiments is most likely due to the broader spectrum of carbon sources used.

The peculiarity of *Pectinatus* spp. exclusively producing the volatiles propanoic acid and acetic acid is confirmed by the literature [5,21,23]. The formation of ethyl propanoate by all studied strains of the genus *Pectinatus* has not been reported so far. In addition to this, our study was able to show that two strains of the species *P. cerevisiiphilus* (DSM 20467^T^, TMW 2.2465) and one *P. haikarae* strain (TMW 2.1496) did not produce acetic acid in contrast to the bulk of strains. This may be due to the lack of a gene or a defective gene needed to produce this compound. α-terpineol, a compound solely present in beer spoiled by *P. frisingensis* strains, is a monoterpenoid alcohol that is widely applied for its aroma attributes. It is also found in beer, and the microbial production during spoilage might be derived from other monoterpenoid hop compounds such as limonene and pinenes [72,73]. *M. sueciensis* also produced this compound in spoiled beer. Nerol is another monoterpenoid alcohol whose level increased in beer spoiled by *P. frisingensis* with the exception of a few strains. Much like α-terpineol, this is another example of a terpenoid alcohol that can be produced by the geraniol metabolism of yeast during fermentation, as well as by beer spoilers during spoilage, as the levels were significantly increased compared with the control [72]. The most unique *Pectinatus* strains proved to be *P. haikarae*, which produced several volatiles that strains from none of the other species were able to produce. This study was able to highlight this species’ ability to produce 2-methylbutyl octanoate, 3-methylbutyl octanoate, 4-vinyl guajacol, methyl-E-geranate, ethyl-4-methyl pentanoate, and methional exclusively, which has not been reported so far. Methional, which is usually associated with aged lager beer [74], surprisingly was only found in beer spoiled by *P. haikarae* and therefore was not produced by aging because of the incubation conditions. The only volatile output that has been reported is propionic and acetic acid from fructose as well as acetoin and H_2_S [21].

The overall notable decrease in pH during beer spoilage by *Pectinatus* spp. originated from the consortium of different acids produced during growth (Figure 2), which aligns well with the literature [5,21,23]. This finding highlights the overall higher acid tolerance of strains from *Pectinatus* spp. in contrast to the *Megasphaera* spp. strains.

**Figure 3:**
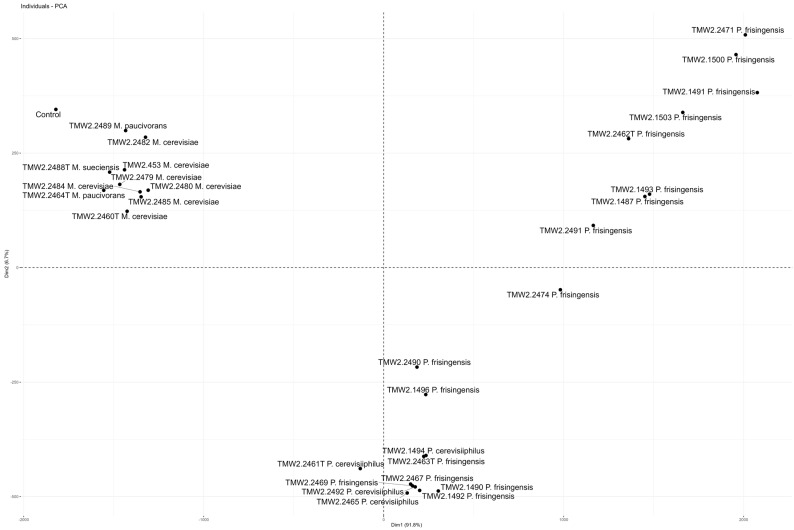
Principal component analysis of the residual carbohydrates in all samples spoiled by the strains as well as in the control beer revealing three main clusters. On the top left, all *Megasphaera* strains cluster in close proximity to the control beer, fortifying the marginal utilization of all carbohydrates by strains of the genus. The bottom cluster harbors most strains of the genus *Pectinatus* that show a significant but similar utilization of glycerol and maltose, whereas the *P. frisingensis* strains in the upper right corner show the highest degradation of maltose and therefore group as a separate cluster. All samples were biological triplicates that were incubated for 23 days at 30 °C, and the control was an unspoiled lager beer treated the same way as the spoiled samples.

In order to distinguish the strains by considering all of their metabolized carbohydrates, a principal component analysis (PCA) was conducted on the residual carbohydrates of all strains (Figure 3).

**Figure 4:**
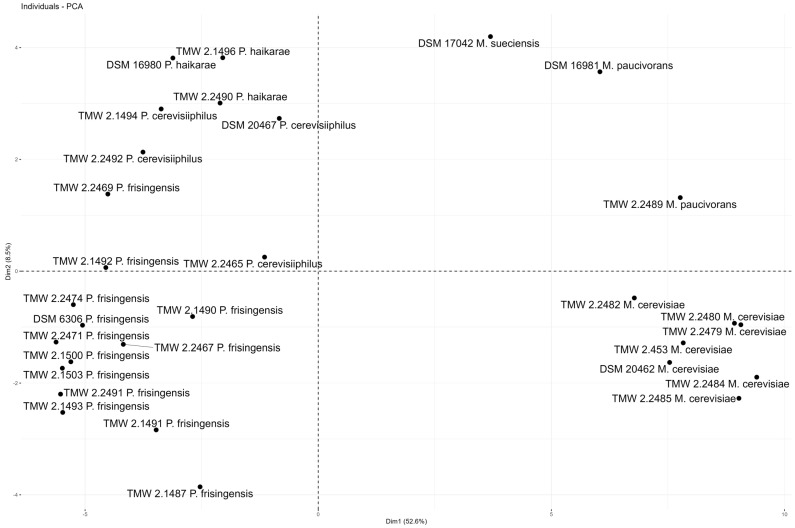
Principal component analysis revealing a genus specificity of the volatilomes of both *Pectinatus* spp. and *Megasphaera* spp. The difference in *Pectinatus* species is caused by the production of organic acids and diverse volatile compounds. All *M. cerevisiae* are distinctly clustered, and *M. paucivorans* and *M. sueciensis* are separated because of a more diverse production of sulfuric compounds. All samples were biological triplicates that were incubated for 23 days at 30 °C, and the control was an unspoiled lager beer treated the same way as the spoiled samples.

The PCA resulted in three distinct clusters, of which one harbors the control as well as all *Megasphaera* strains, coinciding with their observed carbohydrate metabolism. The second cluster on the bottom of the figure incorporates most strains of all three species of the genus *Pectinatus* that show a significant utilization of maltose and glycerol. The *P. frisingensis* strains that show a far higher degradation of maltose in comparison with the other strains of the genus are clustered at the upper right of Figure 3. Another PCA was performed for the produced volatiles (Figure 4). Factoring in all volatiles detected, it revealed a clear separation of the genera. In contrast to the PCA performed with the residual carbohydrates, in this PCA, the *M. cerevisiae* strains cluster, whereas the strains of the other two species (*M. paucivorans*, *M. sueciensis*) dissociate from this cluster based on a more versatile output of sulfuric compounds. Looking at the *Pectinatus* spp. strains in the figure, it becomes apparent that there are also two clusters whose separation is based on the diversity of the organic acids and scents produced. Interestingly, species or strain diversity is more evident in carbohydrate utilization compared with volatilome composition.

## 5. Conclusions

This study is the first to show that the examined strains from the genera *Pectinatus* and *Megasphaera* utilize glycerol and display distinct patterns of carbohydrate utilization as well as volatile production during beer spoilage. This is a novel finding in the field of obligate anaerobic beer spoilers as these microorganisms have been profoundly studied in a laboratory context but to a lesser extent in the beer environment, which is their habitat of origin. We were able to show that the spoilage organisms studied here tolerate beer’s intrinsic antimicrobial barriers and that growth is enabled by a specific adaptation to the substrate. This highlights the importance of extrinsic barriers (brought about by, e.g., mashing, wort boiling, pasteurization, sterile filtration, and cold storage) in beer production as they apparently represent the most effective means of preventing spoilage by *Pectinatus* and *Megasphaera*.

## Figures and Tables

**Table 1:** Overview of the strains used in this study. Superscript T indicates the respective type of strain.

Strain Reference	Genus	Species	Strain	Discovery of Isolate	Date of Sampling
DSM 20462^T^	*Megasphaera*	*cerevisiae*	PAT 1	Spoiled beer, Germany	before 7 June 1979
DSM 20467^T^	*Pectinatus*	*cerevisiiphilus*	CCC B-1022	Spoiled beer, USA	before 2 August 1979
DSM 6306^T^	*Pectinatus*	*frisingensis*	V1	Turbid beer, Finland	1978
DSM 16980^T^	*Pectinatus*	*haikarae*	VTT E-88329	Air of bottling hall in a brewery, Finland	1989
DSM 16981^T^	*Megasphaera*	*paucivorans*	VTT E-032341	Spoiled beer, Italy	2002
DSM 17042^T^	*Megasphaera*	*sueciensis*	VTT E-97791	Spoiled beer, Sweden	1997
TMW 2.2465	*Pectinatus*	*cerevisiiphilus*	33-1	Brewery environment	Unknown
TMW 2.2467	*Pectinatus*	*frisingensis*	HBS 2	Brewery environment	Unknown
TMW 2.2469	*Pectinatus*	*frisingensis*	Mu 2	Brewery environment	Unknown
TMW 2.2471	*Pectinatus*	*frisingensis*	247-2	Brewery conveyor belt	Unknown
TMW 2.2474	*Pectinatus*	*frisingensis*	245-1	Brewery facility table	Unknown
TMW 2.2479	*Megasphaera*	*cerevisiae*	SR V 4	Brewery environment	Unknown
TMW 2.2480	*Megasphaera*	*cerevisiae*	Sp. II 9/4	Brewery environment	Unknown
TMW 2.2482	*Megasphaera*	*cerevisiae*	SR V 5	Brewery environment	Unknown
TMW 2.2484	*Megasphaera*	*cerevisiae*	PAT 2a	Brewery environment	Unknown
TMW 2.2485	*Megasphaera*	*cerevisiae*	PAT 2b	Brewery environment	Unknown
TMW 2.453	*Megasphaera*	*cerevisiae*	M2	Brewery environment	Unknown
TMW 2.1487	*Pectinatus*	*frisingensis*	140	Brewery environment	Unknown
TMW 2.1490	*Pectinatus*	*frisingensis*	173	Brewery environment	Unknown
TMW 2.1491	*Pectinatus*	*frisingensis*	175	Brewery environment	Unknown
TMW 2.1492	*Pectinatus*	*frisingensis*	225	Brewery environment	Unknown
TMW 2.1493	*Pectinatus*	*frisingensis*	227	Brewery environment	Unknown
TMW 2.1494	*Pectinatus*	*cerevisiiphilus*	228	Brewery environment	Unknown
TMW 2.1496	*Pectinatus*	*haikarae*	233	Brewery environment	Unknown
TMW 2.1500	*Pectinatus*	*frisingensis*	240	Brewery environment	Unknown
TMW 2.1503	*Pectinatus*	*frisingensis*	260	Brewery environment	Unknown
TMW 2.2489	*Megasphaera*	*paucivorans*	BEL B	Spoiled beer, Italy	2010
TMW 2.2490	*Pectinatus*	*haikarae*	BIO Y21	Spoiled beer, Finland	2010
TMW 2.2491	*Pectinatus*	*frisingensis*	ABBC437	Brewery environment	Unknown
TMW 2.2492	*Pectinatus*	*cerevisiiphilus*	ABBC474	Brewery environment	Unknown

**Table 2:** Residual carbohydrates and glycerol in biological triplicates of beer spoiled by several species of *Megasphaera* after 23 days at 30 °C. All concentrations are in mg/L. The control consisted of a biological triplicate of lager beer that was incubated at the same conditions. An elevated T indicates the respective type strain. Bold letters indicate that the decrease in the respective carbohydrate was significant. Lit.+ indicates that the source reports degradation of the carbon source. Lit.− indicates that the source reports the species is not able to ferment the carbohydrate. The publications used are a [19] and b [21].

	Species	Glycerol	Arabinose	Xylose	Fructose	Ribose	Maltulose	Maltose	Maltotriose
Control	*-*	1550 ± 45.8	78.8 ± 3.0	82.9 ± 1.6	71.3 ± 2.1	39.8 ± 4.7	1120 ± 12.2	3670 ± 605.6	2130 ± 35.0
DSM 20462^T^	*M. cerevisiae*	**1190**	**0.0**	90.4	**0.0**	**33.4**	1040	3430	2010
TMW 2.453	*M. cerevisiae*	**1280**	**64.4**	89.6	**0.0**	38.6	1040	3400	2030
TMW 2.2479	*M. cerevisiae*	**1250**	69.0	92.6	**0.0**	39.6	1080	3430	2080
TMW 2.2480	*M. cerevisiae*	**1210**	71.5	87.5	**0.0**	42.1	1070	3280	**1930**
TMW 2.2482	*M. cerevisiae*	**1310**	71.0	91.6	77.0	**32.6**	1050	3250	2010
TMW 2.2484	*M. cerevisiae*	**1210**	**18.4**	89.6	**0.0**	41.0	1040	3340	**1983**
TMW 2.2485	*M. cerevisiae*	**1190**	**16.6**	89.1	**0.0**	40.0	1030	3340	**1990**
Lit.+			a		a				
Lit.−		a		a		a		a	
DSM 16981^T^	*M. paucivorans*	**1250**	72.0	94.2	0.0	39.2	1080	3530	2160
TMW 2.2489	*M. paucivorans*	**1360**	72.6	87.0	0.0	39.9	1030	3350	2010
Lit.+									
Lit.−		b	b	b	b	b		b	
DSM 17042^T^	*M. sueciensis*	**1290**	68.6	87.0	**53.3**	**33.3**	1080	3470	2120
Lit.+									
Lit.−		b	b	b	b	b		b	

**Table 3:** Volatiles detected in biological duplicates of beer spoiled by *Megasphaera* spp. strains after 23 days at 30 °C. The analysis was executed using HS-SPME GCMS. The abundance of compounds was determined by statistically comparing the signal of samples in contrast to the control. Lit. indicates that the source reports the production of the compound. An elevated T indicates the respective type strain. An X indicates the presence of the particular compound. The publications used are a [19] and b [21].

	DSM 20462^T^ *M. cerevisiae*	TMW 2.2479 *M. cerevisiae*	TMW 2.2480 *M. cerevisiae*	TMW 2.2482 *M. cerevisiae*	TMW 2.2484 *M. cerevisiae*	TMW 2.2485 *M. cerevisiae*	TMW 2.453 *M. cerevisiae*	Lit.	DSM 16981^T^ *M. paucivorans*	TMW 2.2489 *M. paucivorans*	Lit.	DSM 17042^T^ *M. sueciensis*	Lit.
Dimethyl sulfide	X		X	X					X	X		X	
Propanol		X	X	X	X	X	X			X			
Ethyl acetate						X							
Isobutanol							X					X	
n-Butanol	X	X	X	X	X	X	X		X	X		X	
3-Methylbutanal			X										
2-Methylbutanal			X	X			X						
S-Methyl thioacetate									X	X		X	
Isobutyric acid	X	X	X	X	X	X	X	a		X	b	X	b
Ethyl-Isobutanoate	X	X	X		X	X	X			X			
Butyric acid	X	X	X	X	X	X	X	a	X	X	b	X	b
Ethyl butanoate	X	X	X	X	X	X	X		X	X			
3-Methylbutanoic acid	X	X	X	X	X	X	X		X	X		X	
2-Methylbutanoic acid	X	X	X	X	X	X	X						
Ethyl-2-Methyl butanoate	X	X	X		X	X	X			X			
Ethyl-3-Methyl butanoate	X	X		X	X	X	X						
Furfural	X				X	X	X			X			
Hexanol	X	X	X		X	X	X		X	X			
Styrene					X	X							
Ethyl pentanoate		X	X	X	X	X							
Ethyl furfuryl ether	X	X							X	X		X	
Methional												X	
Dimethyl trisulfide									X	X		X	
Methionol	X	X	X	X	X	X	X			X			
Caproic acid	X	X	X	X	X	X	X	a	X	X	b	X	b
Ethyl hexanoate	X	X	X	X	X	X	X			X			
Hexyl acetate	X	X	X		X	X							
Limonene										X			
Ethyl-5-Methylhexanoate			X		X								
2-Acetyl1H-Pyrrol			X		X								
Octanol	X	X	X		X	X			X	X		X	
Ethyl heptanoate		X	X	X	X	X						X	
2-Nonanol												X	
Heptyl acetate					X								
Methionyl acetate		X											
a-Terpineol												X	
4-Vinylphenol									X	X			
Ethyl nicotinoate	X	X	X	X	X	X	X		X	X		X	
Citronellol									X	X			
Ethyl-2-Phenylacetat		X	X	X	X				X	X			
Decanol									X	X		X	
Geranyl acetate										X			

**Table 4:** Residual carbohydrates and glycerol detected in biological triplicates in beer spoiled by *Pectinatus spp.* strains for 23 days at 30 °C. All concentrations are in mg/L. The type strains are indicated by an elevated T. The control was a triplicate of lager beer that was not inoculated but otherwise treated as the samples. Bold letters indicate that the decrease in the respective carbohydrate was significant. An asterisk indicates a result from duplicates. Lit.+ indicates that the source reports degradation of the carbon source. Lit.− indicates that the source reports the species is not able to ferment the carbohydrate. The publications used are a [5], b [6], and c [21].

	Species	Glycerol	Arabinose	Xylose	Fructose	Ribose	Maltulose	Maltose	Maltotriose
Control	*-*	1550 ± 45.8	78.8 ± 3.0	82.9 ± 1.6	71.3 ± 2.1	39.8 ± 4.7	1120 ± 12.2	3670 ± 605.6	2130 ± 35.0
DSM 20467^T^	*P. cerevisiiphilus*	**69.8**	**0.0**	**0.0**	**0.0**	**29.2**	1020	**2510**	2140
TMW 2.2465	*P. cerevisiiphilus*	**0.0**	**0.0**	**0.0**	**0.0**	41.7	**968**	**2280**	**1790**
TMW 2.1494	*P. cerevisiiphilus*	**83.1**	**64.8**	**0.0**	**0.0**	**36.4**	**965**	**2130**	**1620**
TMW 2.2492	*P. cerevisiiphilus*	**0.0**	**58.3**	**0.0**	**0.0**	**32.9**	**925**	**2240**	**1730**
Lit.+				a		a			
Lit.−								a	
DSM 6306^T^	*P. frisingensis*	**62.9**	**0.0**	103	**0.0**	**0.0**	**637**	**895**	2160
TMW 2.2471	*P. frisingensis*	**0.0**	**0.0**	107	**0.0**	38.3	**356**	**254**	2020
TMW 2.1487	*P. frisingensis*	**0.0**	**18.6**	95.9	**0.0**	**32.4**	**685**	**835**	**1850**
TMW 2.1491	*P. frisingensis*	**0.0**	**18.5**	93.9	**0.0**	34.3	**387**	**207**	**1520**
TMW 2.1493	*P. frisingensis*	**0.0**	**7.63**	98.5	**0.0**	46.2	**657**	**814**	**1810**
TMW 2.1500	*P. frisingensis*	**0.0**	**3.07**	105	**0.0**	**29.5**	**379**	**312**	**1960**
TMW 2.1503	*P. frisingensis*	**0.0**	**3.66**	108	**0.0**	43.8	**525**	**613**	2060
TMW 2.2491	*P. frisingensis*	**81.1**	**0.0**	88.8	**0.0**	37.6	**704**	**1120**	**1790**
TMW 2.2467	*P. frisingensis*	**0.0**	**0.0**	94.6	**0.0**	60.8	**934**	**2260**	**1780**
TMW 2.2469	*P. frisingensis*	**0.0**	**0.0**	96.0	**0.0**	61.1	1050	**2230**	**1830**
TMW 2.2474	*P. frisingensis*	**0.0**	**0.0**	0.0 *	**0.0**	46.0	**792**	**1340**	**1930**
TMW 2.1490	*P. frisingensis*	**0.0**	**8.72**	90.7	**0.0**	57.5	1020	**2090**	**1560**
TMW 2.1492	*P. frisingensis*	**0.0**	**7.33**	95.3	**0.0**	56.5	**766**	**2250**	**1550**
Lit.+		b		a		a			
Lit.−								a	
DSM 16980^T^	*P. haikarae*	**0.0**	**0.0**	**6.37**	**19.5**	38.3	1000	**2160**	**1950**
TMW 2.1496	*P. haikarae*	**136**	81.1	0.0 *	**14.4**	33.6	**968**	**2090**	**1870**
TMW 2.2490	*P. haikarae*	**189**	71.9	137 *	**42.7**	35.5	**925**	**2120**	**1920**
Lit.+		c	c	c	c	c			
Lit.−								c	

**Table 5:** Volatiles present in biological duplicates of beer spoiled by *Pectinatus* spp. strains. The analysis was executed after 23 days at 30 °C using HS-SPME GCMS. The abundance of compounds was determined by statistically comparing the signal of samples with the control. An elevated T indicates the respective type strain. Lit. indicates that the source reports the production of the compound. An X indicates the presence of the particular compound. The publications used are a [5], b [6], and c [21].

	DSM 20467^T^ *P. cerevisiiphilus*	TMW 2.2465 *P. cerevisiiphilus*	TMW 2.1494 *P. cerevisiiphilus*	TMW 2.2492 *P. cerevisiiphilus*	Lit.	DSM 6306^T^ *P. frisingensis*	TMW 2.1487 *P. frisingensis*	TMW 2.1491 *P. frisingensis*	TMW 2.1493 *P. frisingensis*	TMW 2.1500 *P. frisingensis*	TMW 2.1503 *P. frisingensis*	TMW 2.2471 *P. frisingensis*	TMW 2.2474 *P. frisingensis*	TMW 2.2491 *P. frisingensis*	TMW 2.2467 *P. frisingensis*	TMW 2.2469 *P. frisingensis*	TMW 2.1490 *P. frisingensis*	TMW 2.1492 *P. frisingensis*	Lit.	DSM 16980^T^ *P. haikarae*	TMW 2.1496 *P. haikarae*	TMW 2.2490 *P. haikarae*	Lit.
Methyl mercaptan											X												
Dimethyl sulfide						X	X	X	X	X	X	X	X	X	X								
Propanol		X				X																	
Acetic acid			X	X	a, b	X	X	X	X	X	X	X	X	X	X	X	X	X	a, b	X		X	c
Ethyl acetate							X								X								
Isobutanol			X									X			X					X			
3-Methylbutanal												X								X		X	
2-Methylbutanal																				X			
Propanoic acid	X	X	X	X	a, b	X	X	X	X	X	X	X	X	X	X	X	X	X	a, b	X	X	X	c
Ethyl propanoate	X	X	X	X		X	X	X	X	X	X	X	X	X	X	X	X	X		X	X	X	
Propyl acetate		X						X	X	X	X	X	X	X	X								
Isobutyric acid	X	X	X	X		X	X	X	X	X	X	X	X	X	X	X	X	X		X		X	
Isobutyl acetate									X			X		X	X							X	
2,3-Butandiol		X						X	X					X			X	X				X	
Butyric acid	X	X					X	X	X					X	X		X	X				X	
3-Methyl-2-Buten-1-thiol								X	X								X	X					
3-Methyl butyric acid	X	X					X	X							X		X					X	
Furfural		X	X				X	X	X	X	X			X	X		X	X			X	X	
Ethyl furfuryl ether		X				X	X		X	X	X	X		X	X		X						
Methional																				X		X	
Ethyl-4-Methyl pentanoate																						X	
1-Octen-3-ol						X	X	X	X	X	X	X	X	X	X	X							
Methionol	X						X										X						
Caproic acid							X																
Myrcen		X		X		X	X	X	X	X	X	X	X	X	X	X	X	X					
Octanol		X																					
Ethyl heptanoate				X		X	X	X	X	X	X	X	X	X	X	X	X	X					
Linalool												X											
Ethyl-6-Methyl heptanoate													X	X									
a-Terpineol						X	X	X	X	X	X	X	X	X	X	X	X	X					
Nerol						X	X	X	X	X	X	X		X				X					
Geraniol	X	X	X	X		X	X	X	X	X	X	X	X	X	X	X	X	X		X		X	
4-Vinyl guajacol																						X	
Methyl-E-geranate																						X	
Citronellyl acetate		X																					
Ethyl dihydrocinnamate									X														
3-Methylbutyl octanoate																						X	
2-Methylbutyl octanoate																						X	

## Data Availability

The data presented in this study are available on request from the corresponding author due to privacy, legal or ethical reasons.

## Supplementary Materials

The following supporting information can be downloaded at https://www.mdpi.com/article/10.3390/microorganisms12102045/s1, Table S1: Tested compounds to identify the substrates eligible for anaerobic beer spoilage bacteria. All compounds were used in establishing the ion chromatography method eventually used; Table S2: Results from the validation of the carbohydrate analysis; Figure S1: Growth curves of the candidate strains. The diagrams show a growth of all strains and a vast difference between the various species. A superscript T indicates the respective type strain. Strains were anaerobically incubated for 23 days at 30 °C.

## References

[1] Paradh A.D., Mitchell W.J., Hill A.E. (2011). Occurrence of *Pectinatus* and *Megasphaera* in the major UK breweries. J. Inst. Brew..

[2] Matoulkova D., Kosar K., Slaby M., Sigler K. (2013). Occurrence and species distribution of strictly anaerobic bacterium *Pectinatus* in brewery bottling halls. Cerevisia.

[3] Schneiderbanger J., Grammer M., Jacob F., Hutzler M. (2018). Statistical evaluation of beer spoilage bacteria by real-time PCR analyses from 2010 to 2016. J. Inst. Brew..

[4] Goodman M., Neal J.A., Corsi A., Sirsat S.A. (2020). Isolation of beer-spoiling bacteria from Texas craft breweries. J. Culinary Sci. Tech..

[5] Schleifer K.H., Leuteritz M., Weiss N., Ludwig W., Kirchhof G., Seidel-Rüfer H. (1990). Taxonomic study of anaerobic, Gram-negative, rod-shaped bacteria from breweries—Emended description of *P. cerevisiiphilus* and description of *P. frisingensis* sp. nov., *Selenomonas lacticifex* sp. nov., *Zymophilus raffinosivorans* gen. nov., sp. nov., and *Zymophilus paucivorans* sp. nov. Int. J. Syst. Bacteriol..

[6] Tholozan J.L., Membre J.M., Kubaczka M. (1996). Effects of culture conditions on *Pectinatus cerevisiiphilus* and *Pectinatus frisingensis* metabolism: A physiological and statistical approach. J. Appl. Bacteriol..

[7] Tholozan J.-L., Membré J.-M., Grivet J.-P. (1997). Physiology and development of *Pectinatus cerevisiiphilus* and *Pectinatus frisingensis*, two strict anaerobic beer spoilage bacteria. Int. J. Food Microbiol..

[8] Simpson W.J., Smith A.R.W. (1992). Factors affecting antibacterial activity of hop compounds and their derivatives. J. Appl. Bacteriol..

[9] Osman Y.A., Ingram L.O. (1985). Mechanism of ethanol inhibition of fermentation in *Zymomonas mobilis* CP4. J. Bacteriol..

[10] Ingram L.O. (1990). Ethanol tolerance in bacteria. Crit. Rev. Biotechnol..

[11] Hayashi N., Ito M., Horiike S., Taguchi H. (2001). Molecular cloning of a putative divalent-cation transporter gene as a new genetic marker for the identification of *Lactobacillus brevis* strains capable of growing in beer. Appl. Microbiol. Biotechnol..

[12] Sakamoto K., Margolles A., van Veen H.W., Konings W.N. (2001). Hop resistance in the beer spoilage bacterium *Lactobacillus brevis* is mediated by the ATP-binding cassette multidrug transporter HorA. J. Bacteriol..

[13] Booth I.R., Cash P., O’Byrne C. (2002). Sensing and adapting to acid stress. Antonie Van Leeuwenhoek.

[14] Iijima K., Suzuki K., Ozaki K., Yamashita H. (2006). horC confers beer-spoilage ability on hop-sensitive *Lactobacillus brevis* ABBC45cc. J. Appl. Microbiol..

[15] Bergsveinson J., Goerzen S., Redekop A., Zoerb S., Ziola B. (2016). Genetic variability in the hop-tolerance horC gene of beer-spoiling lactic acid bacteria. J. Am. Soc. Brew. Chem..

[16] Narziß L., Back W. (2009). Die Technologie der Würzebereitung, 8. überarb. und erg. Aufl..

[17] Chowdhury I., Watier D., Hornez J.-P. (1995). Variability in survival of *Pectinatus cerevisiiphilus*, strictly anaerobic bacteria, under different oxygen conditions. Anaerobe.

[18] Watier D., Leguerinel I., Hornez J.P., Chowdhury I., Dubourguier H.C. (1995). Heat resistance of *Pectinatus* sp., a beer spoilage anaerobic bacterium. J. Appl. Bacteriol..

[19] Engelmann U., Weiss N. (1985). *Megasphaera cerevisiae* sp. nov.: A new Gram-negative obligately anaerobic coccus isolated from spoiled beer. Syst. Appl. Microbiol..

[20] Marounek M., Fliegrova K., Bartos S. (1989). Metabolism and some characteristics of ruminal strains of *Megasphaera elsdenii*. Appl. Environ. Microb..

[21] Juvonen R., Suihko M.-L. (2006). *Megasphaera paucivorans* sp. nov., *Megasphaera sueciensis* sp. nov. and *Pectinatus haikarae* sp. nov., isolated from brewery samples, and emended description of the genus *Pectinatus*. Int. J. Syst. Evol. Microbiol..

[22] Marchandin H., Juvonen R., Haikara A. (2009). Megasphaera.

[23] Lee S.Y., Mabee M.S., Jangaard N.O. (1978). *Pectinatus*, a New Genus of the Family Bacteroidaceae. Int. J. Syst. Bacteriol..

[24] Zhang W.-W., Fang M.-X., Tan H.-Q., Zhang X.-Q., Wu M., Zhu X.-F. (2012). *Pectinatus brassicae* sp. nov., a Gram-negative, anaerobic bacterium isolated from salty wastewater. Int. J. Syst. Evol. Microbiol..

[25] Caldwell J.M., Juvonen R., Brown J., Breidt F. (2013). *Pectinatus sottacetonis* sp. nov., isolated from a commercial pickle spoilage tank. Int. J. Syst. Evol. Microbiol..

[26] Suzuki K. (2020). Emergence of new spoilage microorganisms in the brewing industry and development of microbiological quality control methods to cope with this phenomenon: A review. J. Am. Soc. Brew. Chem..

[27] Ritter S., Nobis A., Gastl M., Becker T. (2022). Evaluating raffinose family oligosaccharides and their decomposition products by ion chromatography—A method development and advanced repeatability study. Talanta Open.

[28] Schnaitter M., Wimmer A., Kollmannsberger H., Gastl M., Becker T. (2016). Influence of hop harvest date of the ‘Mandarina Bavaria’ hop variety on the sensory evaluation of dry-hopped top-fermented beer. J. Inst. Brew..

[29] Signorell A. DescTools: Tools for Descriptive Statistics 2023. https://cran.r-project.org/package=DescTools.

[30] Makowski D., Lüdecke D., Patil I., Thériault R., Ben-Shachar M.S., Wiernik B.M. (2023). Automated Results Reporting as a Practical Tool to Improve Reproducibility and Methodological Best Practices Adoption. CRAN. https://easystats.github.io/report/.

[31] Wickham H. (2016). ggplot2: Elegant Graphics for Data Analysis.

[32] Galili T., O’Callaghan A., Sidi J., Sievert C. (2018). heatmaply: An R package for creating interactive cluster heatmaps for online publishing. Bioinformatics.

[33] Lemenkova P. (2019). K-means clustering in R libraries cluster and factoextra for grouping oceanographic data. Int. J. Info. Appl. Math..

[34] Otter G.E., Popplewell J.A., Taylor L. (1970). Quantitative chromatography of the oligosaccharides in wort, beer and brewing syrups. J. Chromatogr. A.

[35] Preedy V.R. (2008). Beer in Health and Disease Prevention.

[36] Li M., Du J., Zhang K. (2020). Profiling of carbohydrates in commercial beers and their influence on beer quality. J. Sci. Food Agric..

[37] Powell C.D., Quain D.E., Smart K.A. (2003). The impact of brewing yeast cell age on fermentation performance, attenuation and flocculation. FEMS Yeast Res..

[38] Powell C.D., Diacetis A.N. (2007). Long term serial repitching and the genetic and phenotypic stability of brewer’s yeast. J. Inst. Brew..

[39] Gabriel P., Dienstbier M., Matoulková D., Kosař K., Sigler K. (2008). Optimised acidification power test of yeast vitality and its use in brewing practice. J. Inst. Brew..

[40] Sigler K., Matoulková D., Dienstbier M., Gabriel P. (2009). Net effect of wort osmotic pressure on fermentation course, yeast vitality, beer flavor, and haze. Appl. Microbiol. Biotechnol..

[41] Parker W.E., Richardson P.J. (1970). The quantitative determination of glycerol in beer by gas-liquid chromatography. J. Inst. Brew..

[42] Briggs D.E. (2004). Brewing: Science and Practice.

[43] Langstaff S.A., Guinard J.-X., Lewis M.J. (1991). Instrumental evaluation of the mouthfeel of beer and correlation with sensory evaluation. J. Inst. Brew..

[44] Sohrabvandi S., Mousavi S.M., Razavi S.H., Mortazavian A.M., Rezaei K. (2010). Alcohol-free beer: Methods of production, sensorial defects, and healthful effects. Food Rev. Int..

[45] Albertyn J., Hohmann S., Thevelein J.M., Prior B.A. (1994). GPD1, which encodes glycerol-3-phosphate dehydrogenase, is essential for growth under osmotic stress in *Saccharomyces cerevisiae*, and its expression is regulated by the high-osmolarity glycerol response pathway. Mol. Cell. Biol..

[46] Ansell R., Granath K., Hohmann S., Thevelein J.M., Adler L. (1997). The two isoenzymes for yeast NAD+-dependent glycerol 3-phosphate dehydrogenase encoded by GDP1 and GDP2 have distinct roles in osmoadaption and redox regulation. EMBO J..

[47] Albers E., Larsson C., Lidén G., Niklasson C., Gustafsson L. (1996). Influence of the nitrogen source on *Saccharomyces cerevisiae* anaerobic growth and product formation. Appl. Environ. Microb..

[48] Peat S., Roberts P.J.P., Whelan W.J. (1952). The occurence of fructose in rabbit-liver glycogen. Biochem. J..

[49] Radomski M.W., Smith M.D. (1962). Isolation of maltulose from alpha-amylase hydrolysates of waxy corn starch. Cereal Chem..

[50] Dias F.F., Panchal D.C. (1987). Maltulose formation during saccharification of starch. Starch/Stärke.

[51] Adachi S., Hkuwijitjaru P., Kobayashi T. (2022). Continuous production of maltulose from maltose in a pressurized hot phosphate buffer. Jpn. J. Food Eng..

[52] García-Baños J.L., Villamiel M., Olano A., Rada-Mendoza M. (2004). Study on nonenzymatic browning in cookies, crackers and breakfast cereals by maltulose and furosine determination. J. Cereal Sci..

[53] García-Baños J.L., Corzo N., Sanz M.L., Olano A. (2004). Maltulose and furosine as indicators of quality of pasta products. Food Chem..

[54] Morales V., Olano A., Corzo N. (2004). Ratio of maltose to maltulose and furosine as quality parameters for infant formula. J. Agric. Food Chem..

[55] Clapperton J.F., MacWilliam I.C. (1971). Fermentation of minor wort carbohydrates by brewing yeasts. J. Inst. Brew..

[56] Sugawara S., Nakamura Y., Shimomura T. (1961). Substrate specificity and some properties of crystalline mold maltase. Agric. Biol. Chem..

[57] Pokusaeva K., O’Connell-Motherway M., Zomer A., Fitzgerald G.F., van Sinderen D. (2009). Characterization of two novel alpha-glucosidases from *Bifidobacterium breve* UCC2003. Appl. Environ. Microb..

[58] Zastrow C.R., Hollatz C., de Araujo P.S., Stambuk B.U. (2001). Maltotriose fermentation by *Saccharomyces cerevisiae*. J. Ind. Microbiol. Biotechnol..

[59] Dietvorst J., Londesborough J., Steensma H.Y. (2005). Maltotriose utilization in lager yeast strains: MTT1 encodes a maltotriose transporter. Yeast.

[60] Alves-Jr S.L., Herberts R.A., Hollatz C., Miletti L.C., Stambuk B.U. (2007). Maltose and maltotriose active transport and fermentation by *Saccharomyces cerevisiae*. J. Am. Soc. Brew. Chem..

[61] Bearson S., Bearson B., Foster J.W. (1997). Acid stress responses in enterobacteria. FEMS Microbiol. Lett..

[62] Lund P.A., Tramonti A., Biase D. de. (2014). Coping with low pH: Molecular strategies in neutralophilic bacteria. FEMS Microbiol. Rev..

[63] Guan N., Liu L. (2020). Microbial response to acid stress: Mechanisms and applications. Appl. Microbiol. Biotechnol..

[64] Sandegren E., Enebo L., Guthenberg H., Ljungdahl L. (1954). Studies on amino acids in brewing. ASBCJ.

[65] Gómez-Alonso S., Hermosín-Gutiérrez I., García-Romero E. (2007). Simultaneous HPLC analysis of biogenic amines, amino acids, and ammonium ion as aminoenone derivatives in wine and beer samples. J. Agric. Food Chem..

[66] Kramer T., Kelleher P., van der Meer J., O’Sullivan T., Geertman J.-M.A., Duncan S.H., Flint H.J., Louis P. (2020). Comparative genetic and physiological characterisation of *Pectinatus* species reveals shared tolerance to beer-associated stressors but halotolerance specific to pickle-associated strains. Food Microbiol..

[67] Schwarz P.B., Han J.-Y. (1995). Arabinoxylan content of commercial beers. ASBCJ.

[68] Steiner J., Kupetz M., Becker T. (2023). Advancing quantification of water-extractable arabinoxylan in beer: A high-throughput approach. Polymers.

[69] de Wulf P., Soetaert W., Schwengers D., Vandamme E.J. (1996). D-glucose does not catabolite repress a transketolase-deficient D-ribose-producing *Bacillus subtilis* mutant strain. J. Ind. Microbiol..

[70] Park Y.-C., Choi J.-H., Bennett G.N., Seo J.-H. (2006). Characterization of D-ribose biosynthesis in *Bacillus subtilis* JY200 deficient in transketolase gene. J. Biotechnol..

[71] Park H.-C., Kim Y.-J., Lee C.-W., Rho Y.-T., Kang J., Lee D.-H., Seong Y.-J., Park Y.-C., Lee D., Kim S.-G. (2017). Production of D-ribose by metabolically engineered *Escherichia coli*. Process Biochem..

[72] Haslbeck K., Bub S., von Kamp K., Michel M., Zarnkow M., Hutzler M., Coelhan M. (2018). The influence of brewing yeast strains on monoterpene alcohols and esters contributing to the citrus flavour of beer. J. Inst. Brew..

[73] Sales A., Felipe L., Bicas J.L. (2020). Production, properties, and applications of α-Terpineol. Food Bioprocess Technol..

[74] Da Soares Costa M., Gonçalves C., Ferreira A., Ibsen C., Guedes de Pinho P., Silva Ferreira A.C. (2004). Further insights into the role of methional and phenylacetaldehyde in lager beer flavor stability. J. Agric. Food Chem..

