# Supplementary material for: Carbohydrate Metabolism Differentiates Pectinatus and Megasphaera Species Growing in Beer
Source: Microorganisms. 2024 Oct 10;12(10):2045. doi: 10.3390/microorganisms12102045 (PMC11510336; doi:10.3390/microorganisms12102045)
Supplement: Supplementary file 1 [file microorganisms-12-02045-s001.zip › Arnold et al. 2024 - supplementary materials.pdf]

Article

Carbohydrate Metabolism Differentiates *Pectinatus* and *Megasphaera* Species Growing in Beer

Manuel J. Arnold [1], Stefan W. Ritter [2], Matthias A. Ehrmann [1,*], Yohanes N. Kurniawan [3], Koji Suzuki [3], Thomas M. Becker [2] and Wolfgang Liebl [1,*]

[1] Chair of Microbiology, Technical University of Munich, 85354 Freising, Germany
[2] Chair of Brewing and Beverage Technology, Technical University of Munich, 85354 Freising, Germany
[3] Asahi Quality and Innovations, Ltd., 1-1-21 Midori, Moriya 302-0106, Ibaraki, Japan
* Correspondence: matthias.ehrmann@tum.de (M.A.E.); wliebl@tum.de (W.L.)

Abstract: A Obligate anaerobic beer spoilage bacteria have been a menace for the brewing industry for several decades. Technological advances in the brewing process aimed at suppressing aerobic spoilers gave rise to problems with obligate anaerobes. In previous studies, the metabolic spectrum of *Pectinatus* and *Megasphaera* species has been described, but their metabolism in the beer environment remains largely unknown. We used high performance anion exchange chromatography with pulsed amperometric detection (HPAEC-PAD) and headspace-solid phase microextraction-gas chromatography-mass spectrometry (HS-SPME-GCMS) to further characterize beer spoiled by 30 different strains from the six beer spoiling species of *Pectinatus* and *Megasphaera* (*P. cerevisiiphilus, P. frisingensis, P. haikarae, M. cerevisiae, M. paucivorans and M. sueciensis*). We detected differences in carbohydrate utilization and the volatile organic compounds (volatilome) produced during beer spoilage by all six species. We were able to show that glycerol, one of the basic components of beer, is the common carbon source used by all strains. It appears that this carbon source allows for anaerobic beer spoilage by *Pectinatus* and *Megasphaera* despite the spoilage-preventing intrinsic barriers of beer (iso-α-acids, ethanol, low pH, scarce nutrients), and thus extrinsic countermeasures are key for prevention.

Keywords: beer spoilage; *Pectinatus* spp.; *Megasphaera* spp.; carbohydrate metabolism; volatile fatty acids

---

Table S1: Tested compounds to identify the substrates eligible for anaerobic beer spoilage bacteria. All compounds were used in establishing the ion chromatography method eventually used.

Sugar alcohols	Monosaccharides	Disaccharides	Trisaccharide	Oligosaccharide
Erythritol	Arabinose	Isomaltose	Isomaltotriose	Isomaltotetraose
Glycerol	Fructose	Isomaltulose	Maltotriose	Stachyose
Inositol	Glucose	Kojibiose	Panose	Verbascose
Maltitol	Mannose	Lactose		
Mannitol	Ribose	Maltose		
Sorbitol	Sorbose	Maltulose		
Xylitol	Xylose	Melibiose		
		Nigerose		
		Raffinose		
		Saccharose		
		Trehalose		
		Turanose		
		Xylobiose		

Table S2: Results from the validation of the carbohydrate analysis.

**Carbohydrate**	**linear range [mg/L]**	**R^2 [-]**	**LOD [mg/L]**	**LOQ [mg/L]**	**recovery beer**	**recovery ferment.**
**glucose**	6–263	0.999	0.12	0.36	91 %	92 %
**fructose**	6–257	0.999	0.34	1.03	96 %	92 %
**saccharose**	6–285	1.000	0.75	2.28	88 %	87 %
**maltose**	56–2540	0.998	0.55	1.67	96 %	95 %
**maltotriose**	54–2450	1.000	1.57	4.76	83 %	86 %
**xylose**	6–283	1.000	0.20	0.59	92 %	92 %
**ribose**	6–268	0.999	0.26	0.76	92 %	95 %
**arabinose**	6–283	0.996	0.17	0.51	94 %	94 %
**glycerol**	4–190	0.999	0.11	0.33	93 %	102 %
**maltulose**	6–275	1.000	0.82	2.49	109 %	110 %

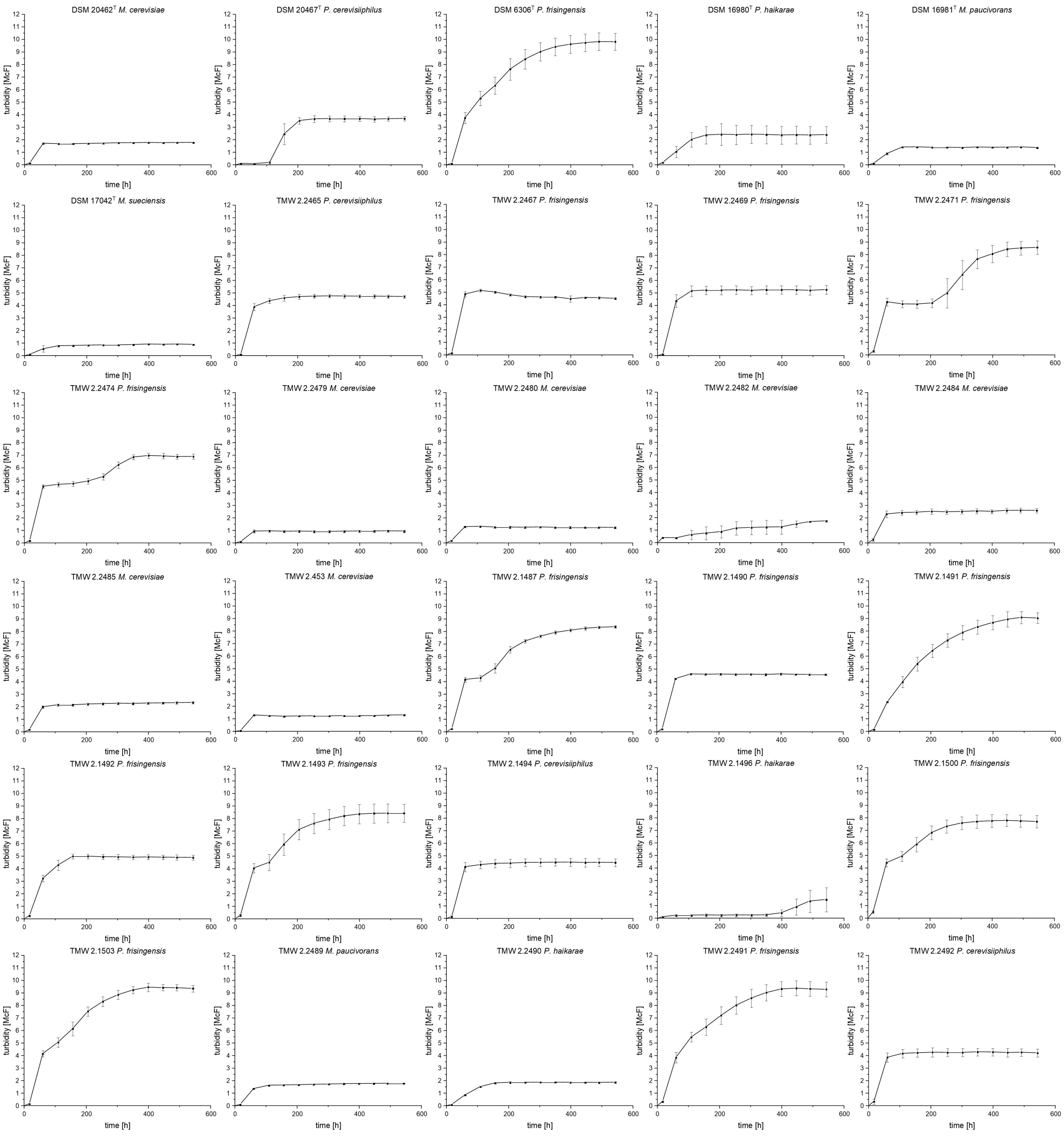

Figure S1: Growth curves of the candidate strains. The diagrams show a growth of all strains and a vast difference between the various species. A superscript T indicates the respective type strain. Strains were anaerobically incubated for 23 days at 30 °C.
